# Bovine Coronavirus Immune Milk Against COVID-19

**DOI:** 10.3389/fimmu.2021.637152

**Published:** 2021-03-23

**Authors:** Antonio Arenas, Carmen Borge, Alfonso Carbonero, Ignacio Garcia-Bocanegra, David Cano-Terriza, Javier Caballero, Antonio Arenas-Montes

**Affiliations:** Department of Animal Health, University of Cordoba, Córdoba, Spain

**Keywords:** BCoV, SARS-CoV-2, immune milk, COVID-19, control

## Abstract

After a year of evolution of the SARS-CoV-2 epidemic, there is still no specific effective treatment for the disease. Although the majority of infected people experience mild disease, some patients develop a serious disease, especially when other pathologies concur. For this reason, it would be very convenient to find pharmacological and immunological mechanisms that help control SARS-CoV-2 infection. Since the COVID-19 and BCoV viruses are very close phylogenetically, different studies demonstrate the existence of cross-immunity as they retain shared epitopes in their structure. As a possible control measure against COVID-19, we propose the use of cow’s milk immune to BCoV. Thus, the antigenic recognition of some highly conserved structures of viral proteins, particularly M and S2, by anti-BCoV antibodies present in milk would cause a total or partial inactivation of SARS-COV-2 (acting as a particular vaccine) and be addressed more easily by GALT’s highly specialized antigen-presenting cells, thus helping the specific immune response.

## Introduction

COVID-19 is a severe human pandemic caused by the SARS-CoV-2 virus. Since there is currently no specific treatment for severe cases, it is critical to find immune mechanisms and strategies to help control the disease. We propose the use of heterologous passive immunity using Bovine Coronavirus immune milk (BIM) as an immunostimulant therapy to control SARS-CoV-2 infection, helping to activate the intestinal immune system.

The coronaviruses were initially classified according to their antigenic characteristics into three serological groups (1), known as Groups 1, 2, and 3, which were later renamed as the new genera *Alphacoronavirus*, *Betacoronavirus*, and *Gammacoronavirus*, respectively (2). Based on their phylogenetic relationships and genomic structures, the *Betacoronavirus* genus now contains five lines or subgenera, including the *Sarbecovirus* (which includes SARS-CoV and SARS-CoV-2), *Merbecovirus* (MERS-CoV), and *Embecovirus* subgenera.

Notable among the embecoviruses are *HCoV-OC43* (OC43), which causes a mild human endemic respiratory infection, and *Bovine Coronavirus* (BCoV). These are two different biotypes of the same species, since the human virus probably evolved from strains of the bovine coronavirus that jumped the species barrier and caused sustained infection in humans (3). BCoV and OC43 share a global nucleotide identity of 96% (4); in contrast, the SARS-CoV-2 genes shared less than 80% nucleotide sequence identity to other Sarbecovirus as SARS-CoV, and about 50% to MERS-CoV (5). Viral seroneutralization techniques show that there is a close antigenic relationship between BCoV and OC43 viruses (6).

Structurally, BCoV (also OC43) is an enveloped virus composed of five structural proteins: the spike glycoprotein (S), the envelope (E) protein, the membrane (M) protein, the nucleocapsid (N) protein, and the hemagglutinin-esterase (HE) protein. The SARS-CoV-2 structure is very similar to the other members of Family Coronaviridae; also contains four structural proteins: S, E, M, and N proteins (5), but it lacks the HE protein.

To attach to host cells, BCoV uses 5-N-acetyl-9-O-acetylneuraminic acid as the preferred receptor to cellular binding (7) whereas SARS-CoV-2 binding angiotensin-converting enzyme receptor (5). Then, the fusion peptide is activated triggering the fusion of viral particle to cellular membrane. The described mechanism allows the virus to infect the host cells.

## Cross-Immune Reactivity Among Betacoronaviruses

The most important immune response generated by coronaviruses is produced against S protein, since it is widely exposed on the viral surface and is an immunodominant structure. S protein is a large class I fusion protein consists of S1 subunit (S1) that contains, among other epitopes the receptor binding domain (RBD), and S2 subunit (S2) that mediates viral membrane fusion (8) contains conserved regions that are necessary for function: the fusion peptide and two conserved repeats (9, 10).

Transmembrane M protein is the most abundant structural protein and is highly conserved among the coronaviruses, but their function is not clearly understood (11). Different M protein epitopes elicit a detectable immune response in the serum of SARS and COVID-19 patients (12, 13). The OC43 M protein is an antagonist of the host antiviral defenses interfering different immune systems (14) and SARS-CoV-2 M protein plays similar effects disabling antiviral signaling cascade (15).

The viral N protein is highly conserved maintaining antigenic cross-reactivity among some coronavirus species, but no between BCoV and SARS-CoV-2 (16). The HE protein, which is not present in other betacoronaviruses (as SARS-CoV-2), enables the BCoV to bind different types of cells. The small E protein is poorly immunogenic for humoral response (17).

Cross-reactivity has been found between OC43 and SARS-CoV (18, 19), which seems to be supported by different antigenic determinants present in N, M, and S2 (a highly conserved region that is almost invariant across the betacoronaviruses), as well as between SARS-CoV and SARS-CoV-2 (20).

SARS-CoV monoclonal antibodies neutralize SARS-CoV-2 through a mechanism, yet unknown, but different from RBD interference. Likewise, alternative mechanisms of coronavirus neutralization by antibodies targeting RBD have been reported, particularly inactivation of the S protein by altering its structure in prefusion conformation (21–23).

A consistent cross-reactivity, but limited cross-neutralization, has been reported between SARS-CoV-2 and other human endemic coronaviruses, including OC43 (24). In addition, recent studies have found that between 40 and 60% of people not previously exposed to SARS-CoV-2 have T helper cells (CD4+) reactive to OC43 (25). This suggests that CD4+ cells specific for endemic betacoronaviruses of the common cold may also recognize the epidemic coronavirus SARS-CoV-2. This hypothesis is reinforced by the seroconversion found in a high proportion (one in three infected) of asymptomatic human SARS-CoV-2 infections (26), some of which could be due to cross-reactivity following exposure to other human coronaviruses (particularly OC43, HCoV-229E, and HECoV viruses).

The formation of immunoglobulin G isotype (IgG) against the RBD domain of the newly emerged SARS-CoV-2 is considered essential for adequate immune protection. These specific IgGs should be absent (or at a low level) in unexposed people, nevertheless, a recent study (27) showed that IgGs reactive to the S2 subunit of SARS-CoV-2 were present in most of the unexposed subjects studied. The authors concluded that cross-reactivity with other human coronaviruses was a plausible explanation. They also found that convalescing COVID-19 patients showed a significant increase in OC43-reactive memory B cells compared with previously unexposed healthy subjects. Consequently, since everything seems to point to strong cross-reactivity between SARS-CoV-2 and OC43 at both the cellular and humoral level, it is likely also to be found with BCoV as reported previously (6). No specific cross-reactivity studies have been performed between SARS-CoV-2 and BCoV, but when the epitope sequence alignment of the SARS-CoV-2 spike proteins was analyzed, a high homology (57 to 83%) with BCoV was found (28).

The phenomenon known as antibody-dependent enhancement (ADE), in which the presence of non-neutralizing antibodies can aggravate the course of the disease, is one of the present general concerns in immunotherapy (and the development of vaccines). The pathophysiology of ADE is not yet well understood, but it appears to be closely related to low levels of neutralizing antibodies against RBD. However, it has been shown that ADE does not occur when antibodies bind to the nucleoprotein or any other structure different from RBD antigens (29).

BCoV causes specific syndromes in cattle with a very high inter- and intra-herd prevalence (30, 31) and has been associated with gastroenteritis and respiratory diseases in lactating calves (32), shipping fever in fattening calves (33) and winter diarrhea in dairy cows (34). Due to their economic, medical, and epidemiological importance, different commercial vaccines against BCoV (including live and inactivated viruses) are currently available and have shown a very good protective effect.

## Immune Milk Against BCoV

Functional foods are products that not only satisfy nutritional requirements but also modulate organic functions and perform highly beneficial tasks in some diseases (35). Bovine milk and colostrum contain high levels of bioactive components, including growth factors, immunoglobulins (Igs), lactoperoxidase, lysozyme, lactoferrin, cytokines, nucleosides, vitamins, peptides, and oligosaccharides, all of them beneficial to human health (36). Hyperimmune cow’s milk has been used against human infections for a long time (37, 38). Raw and pasteurized milk can contain specific antibodies against different human pathogens (39), exerting a synergistic effect on the activity of nonspecific antimicrobial factors (40, 41). Bovine IgG is functionally active throughout the gastrointestinal tract and can prevent digestive and respiratory tract infections in humans (42).

Whole cow’s milk contains more than 30 g/L of protein, almost 1 g/L of which are immunoglobulins, which can reach more than 200 g/L in colostrum (36). IgGs present in bovine milk survive gastric exposure and resist proteolytic digestion in the stomach and intestinal tract, maintaining their active binding capacity to receptors (43). No evidence for the intestinal absorption of intact IgG has been found. In addition, the ingestion of Ig preparations obtained from serum leads to an increase in anti-inflammatory cytokines and a decrease in pro-inflammatory cytokines (44).

It was recently confirmed that SARS-CoV-2 RNA persisted longer (over a month) in the feces of infected people, mainly children, than in respiratory exudates (45, 46). This shows that the infectious period could be prolonged due to the enteric persistence of the virus, even when a person has been considered negative after a routine diagnostic test. We think that the presence of specific IgG in the intestinal lumen would help control viral excretion.

Passive immunity conferred through breast milk has a remarkable protective effect against most of the infectious diseases of animals and has been well studied in almost all coronaviruses of veterinary concern (47–49). The presence of specific antibodies to SARS-CoV-2 has also been found in human breast milk (50).

Ultra-high temperature (UHT) processing is the most widely used heat treatment for the microbiological safety of milk for human consumption, but this treatment destroys the immunoglobulins present in the milk. However, pasteurization is a less aggressive heat treatment that ensures microbiological safety but also preserves most immunoglobulins in milk. Most commercial pasteurized milk from Spain contains antibodies against BCoV, probably due to the high prevalence of infection and the systematic vaccination of cattle. Nevertheless, the heat treatment process used reduces titers below 30. BIM, which we obtained by cow hyperimmunization using commercial coronavirus vaccine, contains antibodies with titers between 128 and 256 (unpublished results) and human consumption could transfer heterologous antibodies against SARS-CoV-2, thus conferring some passive immunity.

## Discussion

The evolution of antibody kinetics after natural infection appears to be very similar between BCoV and SARS-CoV-2. Briefly, after infection, different antibody class (i.e., IgA, IgM, IgG) are produced directed against the different structural proteins of the virus (16). Antibodies directed against RBD are highly neutralizing, but are quite different between the two viruses, as they target different domains. In contrast, antibodies directed towards the M protein may have cross-immunity among coronaviruses, binding selectively to Fc receptors (FcRs) (51). FcRs have interesting immunological functions including phagocytosis, degranulation, antibody-dependent cellular cytotoxicity (ADCC), cytokine formation, lipid mediator, and superoxide production (52).

Animal respiratory coronaviruses often have a pneumoenteric tropism and can persist in carriers who maintain and excrete the virus in respiratory secretions, but mainly in the feces, for long periods of time, even years (30, 53). Fecal excretion has also been demonstrated in SARS-CoV-2 (45, 46) and is even more prolonged than nasal excretion. For BCoV, it has been speculated that, after initial replication, the virus spreads to the gastrointestinal tract by ingestion of large amounts of virus coated in mucous secretions, which would cause the virus to transit to the intestine, causing intestinal infection and subsequent fecal excretion (8).

Gut-associated lymphoid tissue (GALT) (Peyer’s patches, cecal and colonic patches) plays a key role in immunity since it discriminates between food antigens, commensal microorganisms, and pathogens. It should therefore have very safe mechanisms for adjusting immune response to the type of antigen that it locates. These immunoregulatory functions include mechanisms for the recruitment of lymphocytes and myeloid cells from the bloodstream to antigen sampling from surrounding tissue or the surface of the luminal epithelium, as well as others for returning activated effector lymphocytes to the bloodstream or remaining on the mucosal surface where they eliminate pathogens or reside as memory cells.

After consumption of BIM, the ingested antibodies would specifically bind to some shared antigenic structures of SARS-CoV-2. This could totally or partially inactivate the virus and facilitate the processing of SARS-CoV-2 antigens by GALT, but also the formation of immune complexes that can activate the antiviral signaling cascade (15), complement fixation, and binding to the FcRs that control humoral immunity and are essential for an adequate response to infections (52).

Bearing in mind that coronaviruses are markedly pneumoenteric viruses, GALT and other mucosa-associated lymphoid tissues are of enormous importance for the control of infection (54). The reduction in the number of active viral particles in the intestinal mucosa and the activation of other immune mechanisms in the GALT could reinforce the specific immune response against infection by SARS-CoV-2, improving the regulation of the immune system.

Thus, recognition of SARS-CoV-2 by heterologous antibodies in BIM could cause complement fixation of the immune complexes, triggering activation of any of its pathways. This would generate specific responses of neutralization, cytolysis, chemotaxis, opsonization, and others (55), as well as the activation of other immune mechanisms that would benefit immune action, such as the production of IL-10, IFN-β (56), or CD27 cells (57) (which would involve the activation, proliferation, and differentiation of B lymphocytes) or even activation of immunoglobulin class switching (58). The complexity of the cytokine network in humans makes immunomodulation essential in the control of severe COVID-19 and could be achieved by the presence of specific immunoglobulins in the GALT and gut lumen.

Considering the arguments presented above, research should be conducted, focused on exploring the effectiveness of BIM and other immune compounds (milk, milk derivatives, egg yolk, among others) in the control of COVID-19 in human patients.

## Data Availability Statement

The raw data supporting the conclusions of this article will be made available by the authors, without undue reservation.

## Author Contributions

All authors listed have made a substantial, direct, and intellectual contribution to the work and approved it for publication.

## Conflict of Interest

The authors declare that the research was conducted in the absence of any commercial or financial relationships that could be construed as a potential conflict of interest.
